# Prevalence, predictors and reasons for home delivery amongst women of childbearing age in Dodoma Municipality in central Tanzania

**DOI:** 10.4314/ahs.v20i4.52

**Published:** 2020-12

**Authors:** Situ Muhunzi, James Samwel Ngocho, Amasha Mwanamsangu, Leah Sanga, Hellen Hiza, Sia E Msuya, Michael J Mahande

**Affiliations:** 1 Department of Community Health, Institute of Public Health, Kilimanjaro Christian Medical Centre, Moshi, Tanzania; 2 Department of Epidemiology & Biostatistics, Institute of Public Health, Kilimanjaro Christian Medical University College, Moshi, Tanzania; 3 Kilimanjaro Christian Medical University College, Moshi, Tanzania

**Keywords:** Women, home delivery, prevalence, factors, Tanzania

## Abstract

**Introduction:**

The objective was to determine the prevalence, predictors and reasons for home delivery amongst women of childbearing age in Dodoma, Tanzania.

**Methods:**

A cross-sectional study was conducted amongst women living in Dodoma Municipality. Data were collected using adapted questionnaires and analysed using SPPS version 23. A multivariable logistic regression model was used to assess the independent predictors of home delivery.

**Results:**

A total of 425 women of childbearing age were enrolled in this study. The mean (± SD) age of the participants was 28.7 (±7.1) years. The prevalence of home delivery was 35.5% (n=150, 95% CI 30.9 – 40.2). Women with secondary school and above had 93% less odds of home delivery than women who had no education (AOR=0.0795% CI: 0.03–0.18). Women who lived in rural areas (AOR=3.49, 95% CI: 2.12–5.75), and women living more than 5km from health facilities (AOR=2.67, 95% CI: 1.65–4.37) had higher odds of home delivery. The main reasons for home delivery were transportation cost, and long distance to the nearest health facilities.

**Conclusion:**

In this population, the prevalence of home delivery remained to be high. To address this more collaborative multisectoral effort like strengthening health education and strengthening maternity waiting homes are needed.

## Introduction

Home delivery is defined as any birth occurring at home^1^, is considered an unsafe form of delivery and is associated with high risk of maternal and perinatal deaths and other pregnancy complications^2^. These deliveries are generally attended by unskilled birth attendants such as relatives, neighbours and traditional birth attendants^3^,^4^. Globally, the proportion of women delivering at home has declined from 41% in 1990 to 29% in 2015 5. The decline in home delivery was most noted in Southeast Asia and least in sub-Saharan Africa (SSA). In SSA, between 64.9 and 82.4% of births still occur at home^6^.

Complications during pregnancy and unsafe deliveries lead to maternal and neonatal morbidity and mortality, which is a global public health concern^2^,^7^. Globally, an estimated 303,000 women die annually due to pregnancy and child birth complications^8^. The majority (99%) of maternal deaths occur in developing countries^1^,^3^, particularly in SSA. Most maternal deaths occur in rural areas, particularly amongst poor communities^1^,^9^. Furthermore, young adolescents are at a higher risk of experiencing pregnancy and childbirth-related complications than other women^1^. Most of the deaths related to pregnancy and childbirth are preventable.

In Tanzania, according to DHS 2015–16, the maternal mortality ratio reach 556/100,000 live births^5^, higher than what the country had targeted to reach by 2015 (193 deaths per 100,000 live births). In fact, the MMR in Tanzania has remained high since 1990 5. In addition, home births have declined from 49% in 2010 to 37% in 2016 5. And approximately half (46%) of the women in the rural areas of Tanzania deliver at home whereas only 14% of urban women deliver at home There is a wide disparity of home deliveries by region in Tanzania, ranging from 7% in Iringa to 60% in Simiyu 5.

Tanzania has put in place several measures to improve the coverage of skilled birth attendants and lower home delivery rates. Since 2003, maternal and child health services are available free of cost^10^. Efforts to increase the number of health facilities have been enacted with a goal to have one functional dispensary in each village and ahealth centre in each ward. Efforts to strengthen the human resources for health have been employed by increasing the enrolment of nurses and doctors and increasing the number of training institutions and absorption of trained manpower. Furthermore, in-service training on reproductive and child health services to raise the competence of available providers has been expanded. Despite these interventions, the proportion of home deliveries remains unacceptably high, translating to high MMR in the country. In the Dodoma region, 99.2% of women attend their first antenatal visits, but only 69% of pregnant women deliver at health facilities^5^. This is below the national target of 90% by 2015 10. The MMR in Dodoma is 257/100,000 live births^5^. Despite the collaborative efforts from stakeholders, it is not clear why this significant proportion of women still give birth at home. The objective of this study was to determine the prevalence, predictors and reasons for home delivery amongst women of reproductive age in Dodoma Municipality in central Tanzania.

## Methods

### Study area

The Dodoma Municipal Council is one of the eight districts in the Dodoma Region located in central Tanzania and is the capital city of the United Republic of Tanzania. It has a total population of 449,886, of whom 120,935 are women of childbearing age (census report, 2012). The Dodoma Municipal Council is divided administratively into four divisions, which are Zuzu, Hombolo, Dodoma Urban and Kikombo. Down the division is the ward, which is further divided into villages or streets then the hamlet. There are 41 wards, 18 villages, 89 hamlets and 170 streets. The major ethnic groups in the area are Gogo and Rangi, who depend primarily on cultivation, livestock keeping and petty business as their main sources of income and domestic uses. This study involved the following areas: Zuzu and Dodoma Urban.

### Sample size and sampling technique

The sample size was calculated based on the precision and using the previous prevalence of home delivery (46%) from the study performed in 2012 in the Bahi district in Dodoma Region by Lwelamira & Safari^11^. We considered a 5% margin of error and a 10% non-response rate. The single population proportion sample size formula was used to calculate a sample size of 420 for the quantitative study.

This was a community-based cross-sectional study employing both qualitative and quantitative methods. Dodoma Municipality has four divisions, of which one is urban and three are rural. The urban division was purposively selected, and one rural division (Zuzu division) was randomly selected from the three. Within the municipality, sampling was taken proportional to the population size the two-division, the urban division contributed 0.58 of the total sample. A multistage sampling technique was used to select women of childbearing age who delivered two years prior to the study. In the first stage, two wards from each division were randomly selected for a total of four wards (the urban wards were Ntyuka and Chang'ombe and the rural wards were Nala and Chigongwe). From each ward, two streets were randomly selected for a total of 8 streets: Ntyuka, Msembeta, Chihoni, and Chiwondo and Ngh'ambala, Chang'ombe juu, Chilewa and Nyerere. Thereafter, the street executive officer, ten cell leaders and community health workers (CHWs) were used to register the households. The eligible participants were summed and then divided by sample size to reach the nth number of participants. Women who consented were included in the study. We used only one woman per household randomly when we encountered more than one woman who met the inclusion criteria.

### Data collection

The inclusion criteria included women of reproductive age (18 to 49 years) who had lived in the study area for at least one year and who consented by signing the consent form. Those below 18 years we excluded because the study was not approved to enrol minor. A questionnaire in Kiswahili was used for data collection in the quantitative part of the study. The questionnaire included demographic characteristics (age, educational level, economic status, marital status); institutional information (distance from health facility, cost incurred for delivery services, perceived quality of service at the health facility) was also collected. Reproductive information (place of delivery, parity, antenatal clinic (ANC) attendance) was collected as well. Four nurses were trained for two days on the data collection tool and how to collect the data. The interview was administered using the questionnaire distributed by the research assistants.

Focus group discussions (FGDs) using an interview guide were conducted to collect qualitative data for reasons of home delivery. CHWs and hamlet leaders were used to identify the study participants (women of childbearing age who delivered two years before the study) by household registration. Four nurses were trained for two days on the data collection tool and how to collect the data. The interview was administered using the questionnaire distributed by the research assistants. Four FGDs were conducted that included 12 women who delivered at home per group, for a total of 48 participants. During consenting participants were asked if they were willing to take part in the FGD. The list of those who agreed to participate in the FGDs in each ward was used to select the participants. Some women who consented for FGD and were perceived to have rich information according to their age and parity were purposively selected to form one FGD from each ward. Each FGD consisted of 12 participants. The FGD was conducted in the community (at selected classrooms in schools or street executive officers' offices). An interview guide translated in the Kiswahili language was used for the FGD. The main topics discussed included women's perspectives on home delivery, their reasons for home delivery, decision-making on the use of health facility services, and their experience on the topic under discussion. The discussions and interviews were recorded.

### Ethical considerations

The approval to conduct this study was obtained from Kilimanjaro Christian Medical University College Research and Ethics Committee, (certificate number 2051). Permission to conduct the study was also sought from the Dodoma Municipal Director, the Dodoma Municipal Medical Officer of Health and the community leaders of the respective wards, villages and streets. Permission was also sought from the women's respective husbands prior to engaging in the study. Written informed consent was obtained from all participants to whom detailed information of the study was given. Their right to participate in the study or to refuse at any time of the study was explained, and they were assured that all the information obtained from them would only be used for this study and that confidentiality would be observed.

### Data analysis

For the quantitative data, data entry, cleaning and analysis were performed using SPSS version 23 (IBM SPSS Statistics for Windows, Version 23.0. Armonk, NY: IBM Corp.). Descriptive statistics were conducted using frequencies and proportions to summarize the categorical variables. Measures of central tendencies and their respective dispersions were used for continuous variables. A logistic regression analysis was carried out to examine the factors associated with home delivery. The crude odds ratios (cORs) and corresponding 95% CIs were calculated using binary logistic regression. In the bivariable analysis, factors with a p-value ≤ 0.1 were included in the final multivariable model. Additionally, several other variables were selected a priori based on previous studies. Differences with a p-value less than 0.05 were considered statistically significant.

The qualitative data were reviewed daily after each interview to identify any issues that required further information and clarification. All the FGDs recordings were tanscribed and translated in English. For each transcription, issues relating to the study aim were identified and coded. After the completion of coding, themes were developed and classified, guided by the questions used, such as women's perspectives on home delivery, reasons for home delivery and decision-making regarding the use of health facility services.

## Results

The study team approached 425 women, and all 425 agreed to participate, giving a response rate of 100%. Two women had missing data and were excluded, leaving 423 in the analysis. The mean age of the participants was 28.7 (± SD 7.1) years. Of the 423 participants, the majority were married (86%), had primary or higher education (70%) and had two or more children (77%) (Table 1). Seventy percent of the 423 women reported that the distance to the nearest health facility was < 5 km (figure 2). Nearly 58% of the 423 women reported that during their last pregnancy, they attended the ANC for four or more visits as recommended. The majority (87%) participated in making decisions regarding where to deliver their babies and received health service either alone or jointly with their partner (Table 1).

**Table 1:** Background characteristics amongst women of reproductive age in Dodoma (n=423)

Variable	n	%
Maternal age (years)		
<20	52	12.3
20–35	284	67.1
>35	87	20.5
Marital status		
Married	363	85.8
Not married	60	14.2
Education level		
None	124	29.3
Primary	238	56.2
Secondary or above	59	13.9
Missing	2	0.5
Occupation		
Subsistence farmer	227	53.8
Housewife	112	26.5
Self-employment	50	11.8
Formal employment	33	7.8
Transport cost perception		
High	175	41.4
Moderate	31	7.3
Normal	185	43.7
Low	32	7.6
Parity		
Primipara (1)	97	22.9
Multipara (2–4)	235	55.5
Grand multipara (≥.5)	91	21.6
ANC visits		
None	3	0.7
<4	174	41.1
≥4	246	58.2
Decision-making		
Myself	134	31.7
Husband/Partner alone	46	10.8
Myself + husband/partner	233	55.1
Others	10	2.3
Delivery problem at last pregnancy		8.0
Excessive bleeding	34	
Retained placenta	18	4.3
Loss of a baby	10	2.3
Fits	1	0.2
None	360	85.1
Distance to nearest health facility		
≤5 km	298	70.4
>5 km	125	29.6
Place of delivery at last pregnancy		
Health facility	273	64.5
Home	150	35.5
Residence		
Urban	248	58.6
Rural	175	41.4
Perception of cost delivery		
Normal	3	0.7
Low	163	38.5
High	257	60.8

Of the 423 women, 150 delivered at home during their last birth, for a home birth prevalence of 35.5% (95% CI 30.9 – 40.2). In the bivariable analysis, level of education, occupation, parity, number of ANC visits, distance, decision-making and area of residence were found to be significantly associated with home delivery (table 2).

**Table 2:** Factors associated with home delivery (n=423)

Characteristics	Total	Home delivery	COR [95% CI]	AOR [95% CI]
**Maternal age (years)**				
<20	52	17 (32.7)	0.83 [0.44–1.55]	0.66 [0.33–1.31]
20–35	284	105 (37.0)	1.0	1.0
>35	87	28 (32.2)	0.81 [0.49–1.35]	0.77 [0.44–1.34]
**Education level**				
None	124	73 (58.9)	1.0	1.0
Primary	238	71 (29.8)	0.30 [0.19–0.47]	0.30 [0.19–0.48]
Secondary	59	6 (10.2)	0.08 [0.03–0.20]	0.07 [0.03–0.18]
**Occupation**				
Unemployed	389	139 (35.8)	1.0	1.0
Employed	33	11 (33.3)	0.90 [0.42–1.91]	1.11 [0.48–2.59]
**Marital status**				
Married	363	123 (33.7)	1.0	1.0
Unmarried	60	27 (45.0)	1.60 [0.92–2.78]	2.60 [1.33–5.11]
**Decision maker**				
Wife	134	41 (30.4)	1.0	1.0
Husband	46	25 (54.5)	2.70 [1.36–5.36]	2.88 [1.32–6.27]
Wife & husband	233	80 (34.3)	1.19 [0.75–1.87]	1.84 [1.08–3.15]
Others	10	4 (40.0)	1.51 [0.41–5.65]	2.01 [0.46–8.81]
**Area of residence**				
Urban	248	54 (21.8)	1.0	1.0
Rural	175	96 (54.9)	4.37 [2.86–6.67]	3.49 [2.12–5.75]
**Parity**				
Primipara	97	18 (18.6)	1.0	1.0
Multipara	235	80 (34.0)	2.27 [1.27–4.04]	2.14 [1.20–3.84]
Grand multipara	91	52 (57.1)	5.85 [3.03–11.31]	5.30 [2.73–10.30]
**ANC visits**				
<4	174	75 (42.4)	1.0	1.0
≥4	246	73 (29.7)	0.56 [0.37–0.84]	0.61 [0.40–0.94]
**Distance to HF**				
<5 km	298	83 (27.7)	1.0	1.0
>5 km	125	67 (53.6)	2.99 [1.94–4.61]	2.67 [1.65–4.31]
**Perception of cost** **delivery**				
Normal	244	65 (26.6)	1.0	1.0
Low	22	1 (4.6)	0.13 [0.02–0.99]	0.13 [0.02–0.99]
High	157	84 (53.5)	3.17 [2.08–4.83]	2.93 [1.88–4.56]

COR: Crude odds ratio; AOR: Adjusted odds ratio; CI: confidence interval

Adjusted for maternal age, education level, occupation, marital status, decision maker, area of residence, distance to health facility, ANC visits, parity and perceived delivery cost

In the multivariable logistic regression analysis, education level, decision-making, parity, ANC visits, area of residence and distance to the nearest health facility remained independent predictors of home delivery (Table 2). Women with secondary and above education had 93% significantly lower odds (AOR 0.07, 95% CI: 0.03–0.18) of delivering at home than those with no education. Women whose partner decided where to deliver their babies had approximately 3 times higher odds (AOR 2.88, 95% CI: 1.32–6.27) of delivering at home than others. Women with more than five children still had 5 times higher odds of delivering at home than their counterparts with fewer children (AOR 5.3, 95%CI: 2.73 -10.30). Women who attended an ANC for four or more visits had 39% significantly lower odds (AOR 0.61, 95% CI: 0.40–0.94) of delivering at home than those with less than four visits. However, women who lived ≥5 km were almost three times as likely (AOR 2.67, 95% CI: 1.65–4.31) to deliver at home than those who lived ≤5 km to the nearest health facility. Women who resided in rural areas had 3 times higher odds of home delivery.

Four FGDs were conducted involving a total of 48 participants, 12 in each group, with an age range of 16–49 years. The main reasons for home delivery were transportation cost, inaccessibility of health facility, long distance to the nearest health facilities and sudden onset of labour/night labour. The available means of transportation for a woman to reach a health facility is either by foot or motor vehicle, which is not convenient to transport pregnant women in labour. Public transport is not available at all times and is absent in some areas.

“*If labour comes suddenly and health facilities are far, the only choice of transport is on foot, which is not comfortable to anyone let alone to a woman in labour*,” said a 32-year-old FGD participant from Chiwondo village.

Another woman (42 years old) from Ngh'ambala village said “*The problem of remote areas is geographical challenge which threatens the lives of expectant mothers*.”

Other reasons mentioned by the participants were poor service and bad language used by health care workers at the health facility that deter women from seeking delivery service care from health facilities. Poor interaction between medical staff and the pregnant mother, particularly when they are in labour, can influence the maternal choice on place of delivery. The majority of women preferred services from male staff rather than female staff. One lady (36 yrs old) from Msembeta village said: “*In my previous delivery, as I approached pushing my baby and trying to call a nurse (female nurse) for help, she answered me to wait while she put on gloves while putting them on very slowly*.” She added: “*Could you keep quiet because nobody told you to close your windows and doors at home. As a result, I delivered alone and she only came to cut off the cord*.”

Cultural practice also contributes to home delivery. Pregnant women in their first pregnancy are often required by tradition to deliver at their mother's house. Moreover, those who have had several deliveries are discouraged from going to facilities to be delivered by young nurses. They believe that grand multiparous deliveries in health facilities are weak.

“*For the first pregnancy, you should come back home and deliver at your mother's house, but from the second pregnancy onwards you could deliver anywhere either hospital or home*” said one participant from Chihoni village (a 47-year-old woman).

Another reason given by the participants was that their husband and his family members decide on a place of delivery. In those households where the head of the family is the husband, he is the one who decides where his wife should deliver.

“*My husband has to decide where I should go for delivery because he is in charge of the house; he took me from my parents and he is the one who has money to pay for costs, either in hospital or at Mdala's houses*” (an old woman who used to help them for delivery), said a young lady of 19 yrs from Chang'ombe street.

## Discussion

The results of this study show that approximately onethird of the women in Dodoma deliver at home. Home delivery was significantly associated with level of education, living far from a health facility, ANC visits attended, area of residence and the decision maker of the place of delivery. Poor services and abusive language of health care providers, cost and inaccessibility of transport, distance to the nearest health facility, sudden onset of labour and culture were amongst the strong reasons reported to influence home delivery.

The prevalence of home delivery in Dodoma is unacceptably high and remains behind the Millennium Development Goal (MDG) target of <10% home deliveries as stipulated by MDG 5 12. The prevalence, however, is reltively lower than that reported in the neighbouring district of Bahi (46%) 11 and Biharamulo District (44%) 3. The lower prevalence in our study might be because most people in Dodoma Municipality live close to the nearest health care facilities that provide delivery services. Elsewhere in East Africa, what we found can be considered high. For example, in Rwanda, the prevalence of home delivery was estimated to be around 7% 13. In East Africa, Rwanda has the lowest proportion of home delivery. The Rwanda success story is partially due to their improvement in the accessibility of maternal care. Additionally, they use CHWs to identify pregnant women and encourage them to deliver at the facility 14.

The person who had decision-making power was found to be significantly associated with home delivery. This finding agrees with studies elsewhere in Tanzania 3 and Ethiopia 15,16. The probable explanation on lack of decision-making power amongst women in their family might be due to society values in the area, which give decision-making power to the husband. In many rural parts of Tanzania, women's power to make decisions is limited, even over matters directly related to their own health, particularly reproductive health 15. From our qualitative and another study from Zambia 4, traditionally men are powered with the final decision of where the woman should deliver since they are the head of the household. Women living in rural areas had higher odds of delivering at home, similar to studies in Ethiopia and Kenya^15^,^17^. This could be explained by physical access to health services between the rural and urban: those living in rural areas have little access to health services – either from living far or having challenges in accessibility of transport to reach the facility.

In this study, the number of ANC visits was a predictor of home delivery. The World Health Organization has set a minimum of four visits for every pregnant woman. Mothers with four or more ANC visits were less likely to deliver at home when compared to those with less than four ANC visits. Findings are similar to other studies in Ethiopia, Ghana, and Bangladesh^18^–^20^ which found that those who had at least four or more antenatal consultations were less likely to deliver at home compared to those with less than four ANC visits. A possible explanation could be that women who attend ANCs more frequently are more likely to receive counselling and health education messages on pregnancy and safe delivery.

In this study, responders who lived ≥5 km were more likely to deliver at home when compared to those who lived close to the nearest health facility. The results are consistent with the evidence from a recent review which found that having access to obstetric care within 5 kilometres was associated with facility delivery^21^. These findings might be attributed to the fact that shorter distances minimize the time to reach the health facility or may reduce the cost of transportation to reach the service. Similar findings have been reported from elsewhere in Tanzania, Kenya and Malawi^11^,^22^–^24^.

Supported by a recent systematic review of barriers to obstetric care at facilities in sub-Saharan Africa^25^. This study found that the majority of women who delivered at home reported the distance to health facilities and poor services (including abusive language from the health care workers) to be their main reasons for delivering at home. Other reasons were sudden onset of labour, transport cost and inaccessibility, culture and partner decision on the place of delivery. Similar reasons have been described by other investigators^26^,^27^. All these reasons may pose a great danger to the women and their babies. In general, relatives, mothers and mothers-in-law attend the women giving birth; however, few of them are attended by traditional birth attendants who are likely unskilled.

The study findings are likely to have some limitations in their interpretations. This may be due to recall bias arising from the study population used and geographical bias because it was difficult to reach the participants in some of the study villages since households were dispersed from one another. Information bias may have risen from fear of health care workers, which may lead to little information from the participants, particularly during the FGDs. Risk of sampling error, though a multistage sampling technique a design effect considered in sample size calculation to minimize this error.

## Conclusion

The prevalence of home delivery in Dodoma is high. Predictors influencing home delivery amongst mothers include educational level, more than five deliveries, distance to health facilities, less than four ANC visits and poor health care services. The main reasons for home delivery were transportation cost, long distance to the nearest health facilities and sudden onset of labour. Interventions should target all the predictors and reasons obtained to increase facility deliveries and overcome the dangers imposed to women and their babies during home delivery.

## Figures and Tables

**Table 3:** Reasons for home delivery

Reason	n	%
Poor service provided by health care givers	41	85.4
Distance to the nearest health facility	40	83.3
Transport cost and accessibility	36	75.0
Husband's decision-making on place of delivery	31	64.6
Cultural practices	20	41.7
Sudden onset of labour/ night labour	18	37.5

## Declarations

### List of Abbreviations

ANC-Antenatal Care Visits, FGD-Focus Group Discussion, MOHSW-Ministry of Health and Social Welfare, MoHCDGEC -Ministry of Health Community Development Gender Elderly and Children, SD-Standard Deviation, SSA-sub Saharan Africa, WHO-World Health Organization.

### Availability of data and material

The datasets analysed during the current study are not publicly available to protect the participants' anonymity but are available from the corresponding author on reasonable request.
